# Diffusion tensor tractography of the mammillothalamic tract in the human brain using a high spatial resolution DTI technique

**DOI:** 10.1038/s41598-018-23452-w

**Published:** 2018-03-27

**Authors:** Arash Kamali, Caroline C. Zhang, Roy F. Riascos, Nitin Tandon, Eliana E. Bonafante-Mejia, Rajan Patel, John A. Lincoln, Pejman Rabiei, Laura Ocasio, Kyan Younes, Khader M. Hasan

**Affiliations:** 1 Department of Diagnostic Radiology, University of Texas Health Science Center at Houston, Houston, Texas USA; 2 University of Texas Health Science Center at Houston, Medical School, Houston, Texas USA; 3 Department of Neurosurgery, University of Texas Health Science Center at Houston, Houston, Texas USA; 4 Department of Neurology, University of Texas Health Science Center at Houston, Houston, Texas USA

## Abstract

The mammillary bodies as part of the hypothalamic nuclei are in the central limbic circuitry of the human brain. The mammillary bodies are shown to be directly or indirectly connected to the amygdala, hippocampus, and thalami as the major gray matter structures of the human limbic system. Although it is not primarily considered as part of the human limbic system, the thalamus is shown to be involved in many limbic functions of the human brain. The major direct connection of the thalami with the hypothalamic nuclei is known to be through the mammillothalamic tract. Given the crucial role of the mammillothalamic tracts in memory functions, diffusion tensor imaging may be helpful in better visualizing the surgical anatomy of this pathway noninvasively. This study aimed to investigate the utility of high spatial resolution diffusion tensor tractography for mapping the trajectory of the mammillothalamic tract in the human brain. Fifteen healthy adults were studied after obtaining written informed consent. We used high spatial resolution diffusion tensor imaging data at 3.0 T. We delineated, for the first time, the detailed trajectory of the mammillothalamic tract of the human brain using deterministic diffusion tensor tractography.

## Introduction

Limbic system of the human brain is a collection of gray and white matter structures that supports a variety of functions including emotion, behavior, motivation, long-term memory, and olfaction. The human thalamus “the bed of the brain” is at the central connections of numerous brain pathways and even though it’s not primarily considered as part of the human limbic system, the thalamus is shown to be involved in many limbic functions of the human brain^1^. The thalamus is connected to many cortical areas involved in limbic system such as the prefrontal cortex and has a critical role in the fronto-limbic circuitry and integrates many interconnecting tracts between components of the limbic system. The major connections of the anterior thalamic nuclei with the hypothalamic nuclei are known to be through the mammillo-thalamic tracts (MTT) which run vertically from the mammillary bodies to the anterior thalamic nuclei^1^. As a vital connection between the mammillary body and anterior thalamus, the MTT is crucial for normal memory functions^2,3^. Studies and case reports on animals indicate that lesions of the MTT cause deficits in retention of conditioned avoidance behavior, in acquisition of new cognitive skills, and in encoding visuo-spatial working memory, as well as deficits in recent memory^2,4–8^. Investigations of MTT lesions in patients have shown that damage to the MTT leads to cognitive impairments in Korsakoff syndrome and Wernicke’s encephalopathy in addition to possibly causing diencephalic amnesia^9,10^. Diencephalic amnesia has been most consistently reported being associated with pathology in the mammillary bodies, the mammillothalamic tract and the anterior thalamic nuclei^2,11–14^. Furthermore, studies suggest that MTT damage after thalamic infarct is the only predictor of whether amnesia will develop in patients^2,3,15^. Lastly, besides its role in memory, the MTT could possibly be involved in the propagation and initiation of generalized seizures^16^. While ample evidence points to the fundamental role of the mammillary bodies as part of the hypothalamic nuclei in central limbic circuitry, the neural circuits underlying these structures are not characterized or incompletely described in the human brain on prior diffusion tensor tractography (DTI) studies. In our most recent DTI studies, we demonstrated for the first time, the direct connections of the amygdala with the hypothalamic and septal nuclei via the amygdalofugal tract^17^. We also demonstrated the indirect connections of the mammillary bodies to the amygdala through the fornix and hippocampus^18^.

Diffusion tensor tractography (DTT) is a robust technique based on diffusion tensor imaging which allows noninvasive vivo reconstruction of the trajectory of the neuronal fiber tracts^19^. This technique may provide information about the course, integrity, anatomical connectivity, or possible disruption of neural pathways. DTT may be helpful in better visualizing the surgical anatomy of the brain structures, in assisting to plan surgical procedures to avoid damaging the important structures, and in exploring the specific connections that are impaired.

Numerous anatomical details in the brain white matter connectivity including the delicate limbic structures have been undetectable or poorly detectable in prior DTT studies due to poor spatial resolution, low signal-to-noise ratio (SNR), and partial volume averaging upon using large voxel volumes^20–23^. For example, the major thalamo-limbic connections of human limbic system including the mammillothalamic tract have not been depicted in the prior DTT studies. This work aimed to present for the first time the trajectory, connectivity, and descriptive anatomy of the mammillothalamic tract (MTT) in the human limbic system using a high resolution DTI protocol on 3 T and deterministic tractography approach^24^.

## Materials and Methods

### Study Subjects

Fifteen healthy adults (3 females and 10 males age range 24–37 years) were included in this study, after obtaining written informed consent. The exclusion factor for our study group was the knowledge of any prior brain pathology including the traumatic, neoplastic, demyelinating disease, or degenerative diseases such as Alzheimer or dementia. The rationale behind our exclusion criteria was to seek the existence of the fine neuronal bundle tract of the MTT in the normal human brain as a preliminary report. Brain MRI scans were also visited by a neuroradiologist to ensure the absence of any intracranial abnormality. This work was approved by our institutional review board (IRB) and was health insurance portability and accountability act (HIPAA) compliant Committee for the Protection of Human Subjects Office https://www.uth.edu/cphs/contact-cphs.htm.

### Conventional MRI Data Acquisition

All MRI studies were performed on a 3 T Philips Intera scanner with a dual quasar gradient system with a maximum gradient amplitude of 80 mT/m, maximum slew rate 200 mT/ms/m, and an eight channel SENSE-compatible head coil (Philips Medical Systems, Best, Netherlands). The conventional MRI (cMRI) protocol included axially prescribed 3D spoiled gradient (repetition time, TR = 8 ms; echo time, TE = 4 ms; and flip angle = TR/TE/flip angle = 8 ms/4 ms/6°), 3-D proton density-weighted (TR/TE/flip angle = 10,000 ms/10 ms/90° and 3-D T_2_-weighted (TR/TE/flip angle = 10,000 ms/60 ms/90°), with a square field-of-view (FOV) = 256 mm × 256 mm and a matrix of 256 × 256 pixels. The slice thickness for the MRI sequences was 1.0 mm with 120 contiguous axial slices covering the entire brain (foramen magnum to vertex). In brief, using a 3 T MRI clinical scanner equipped with state-of-the-art gradient hardware and parallel imaging, it was impossible to collect the ideal isotropic 1 × 1 × 1 mm data set to match exactly the high resolution T1w data. The data were acquired at approximately 1 × 1 × 2 covering the entire human brain keeping an eye on TE and TR so that 120 slices are acquired in a single-shot EPI and using one slap. We have repeated this paradigm three times to match the SNR level attained using the 3 mm and 2 mm protocol (e.g. 120 = 3*40 or 12–60*2 mm). A true 1 mmx1mm in plane acquisition would have resulted in 1/4 the SNR level which would have required 16 times the number of averages to meet the working SNR in the current protocol (i.e. SNR(b0) ~ dx*dy*thickness*sqrt(NEX)). Our data is k-space interpolated. We have acquired true 1 × 1 × 1mm data sets but using multiple slab acquisitions that required all slabs to be realigned if whole brain tractography is sought, but the SNR in this acquisition was poor and we applied special “non-linear” filtering procedures. The current protocol is more time-efficient for all possible fiber tracts and warrants easier clinical translation. The time of scan in our protocol was about 20 minutes which is affordable not only for normal adults but also for patients. We acquired axial sections covering the entire brain as it is done routinely on patients. The selection of b = 500 also reduced signal losses and geometric distortions while providing minimum echo time to cover 120 slices in one shot and one slab for the full brain coverage.

### DTI Data Acquisition

Diffusion-weighted image (DWI) data were acquired axially from the same graphically prescribed cMRI volumes using a single-shot multi-slice 2D spin-echo diffusion sensitized and fat-suppressed echo planar imaging (EPI) sequence, with the balanced Icosa21 tensor encoding scheme^24^. The b-factor = 500 sec mm^−2^, TR/TE = 14460/60 msec. The spatial coverage for DTI data matched the 3D cMRI spatial coverage (FOV = 256 mm × 256 mm and slice thickness/gap/#slices = 1 mm/0 mm/120). The EPI phase encoding used a SENSE k-space undersampling factor of two, with an effective k-space matrix of 112 × 112 and an image matrix after zero-filling of 256 × 256. The acquisition spatial resolution for DTI data was ~ 2.29 mm × 2.29 mm × 1 mm, and the nominal resolution after image construction was 1 mm × 1 mm × 1 mm. The number of b-factor ~ 0 (b_0_) magnitude image averages was four. The total DTI acquisition time was ~ seven minutes for the diffusion-weighted acquisition. The DTI acquisition was repeated three times to enhance the signal-to-noise ratio (SNR). The selection of the b-factor, parallel imaging, repetition and echo times enabled entire brain coverage using single-shot and interleaved EPI. The thin slice acquisition in space and replication of data in time combined with the DTI encoding provided several quality control options to study signal-to-noise ratio and partial volume effects on the DTI tracking results^24^. All methods were performed in accordance with the relevant guidelines and regulations.

### White Matter Fiber Tracking

All DTI data were inspected and coregistered to the reference b = 0 volume then the three scans were averaged to enhance SNR. Geometric eddy current distortions were corrected using a mutual information method implemented by the Philips Pride system. Processing of data including tensor decoding, diagonalization or eigenvector and eigenvalue estimation and scalar map estimation (i.e FA, MD) were conducted in DTIstudio (Johns Hopkins University, Baltimore, Maryland; http://cmrm.med.jhmi.edu/) which was also used for tractography. The WM fiber tracking was performed using DTI Studio software. Fiber tracking was based on the fiber assignment by continuous tracking (FACT) algorithm with a fractional anisotropy (FA) threshold of 0.22 and angle threshold of 60 degrees. The tractography was performed by two tractography experts (AK and KH) with more than 10-years experience in DTI tractography. Reproducibility of the fiber construction in both hemispheres was tested by two experienced raters in all subjects. No significant interrater variability was identified between the two raters.

**Figure 1:**
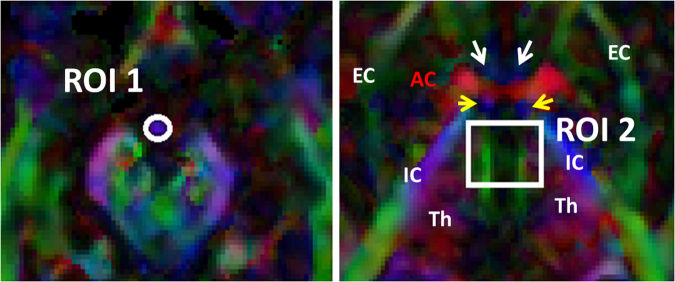
ROI locations for tractography of the MTT. The first ROI (1) was selected over the vertical blue fibers of the mammillary bodies (ROI1). The second ROI (2) was selected on the axial level through anterior commissure (AC). The second ROI was added on the fibers generated in the region of the anterior thalamic nuclei (ROI2). Adding the second ROI by an AND operation will exclude possible contamination from the forniceal columns and stria terminalis due to close proximity of these structures with the MTT. The white arrow heads point at the precommissural fibers of the forniceal columns. The yellow arrow heads point to the postcommissural fibers of the fornix and stria terminalis. AC = anterior commissure; EC = external capsule; IC = internal capsule; Th = thalamus.

Two ROIs were applied to obtain each fiber tract (Fig. 1) and an “AND” operation was performed to include the fibers passing through both ROIs. The first ROI (1) was selected over the vertical blue fibers of the mammillary bodies on the axial plane coursing through the mammillary bodies (Fig. 1, ROI 1). The second ROI (2) was selected on the axial level passing through the anterior commissure (AC). The second ROI was placed on the fibers generated in the region of the medial aspect of the anterior thalamic nuclei (Fig. 1, ROI 2). Adding the second ROI by an AND operation will exclude possible contamination from the columns of the fornix and stria terminalis due to close proximity of these structures with the MTT.

## Results

**Figure 2:**
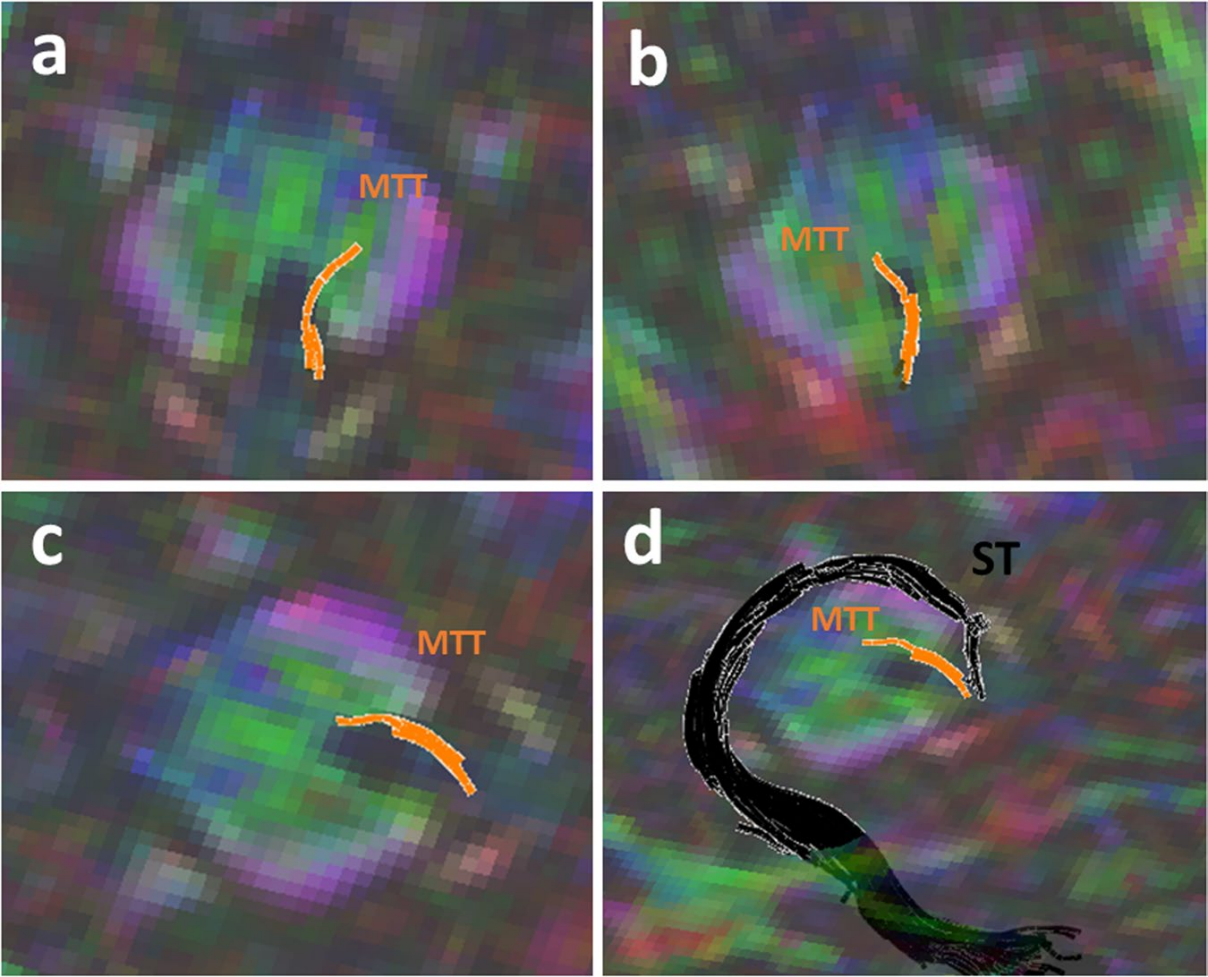
Three-dimensional reconstructions of the MTT (in orange) and the stria terminalis (ST, in black, **d**). Lateral curvature of the MTT is shown within the anterior thalamic nuclei on both sides (**a**,**b**). As illustrated in Fig. 1d, the stria terminalis is located more anteriorly to the MTT and terminates in the anterior hypothalamic nuclei.

**Figure 3:**
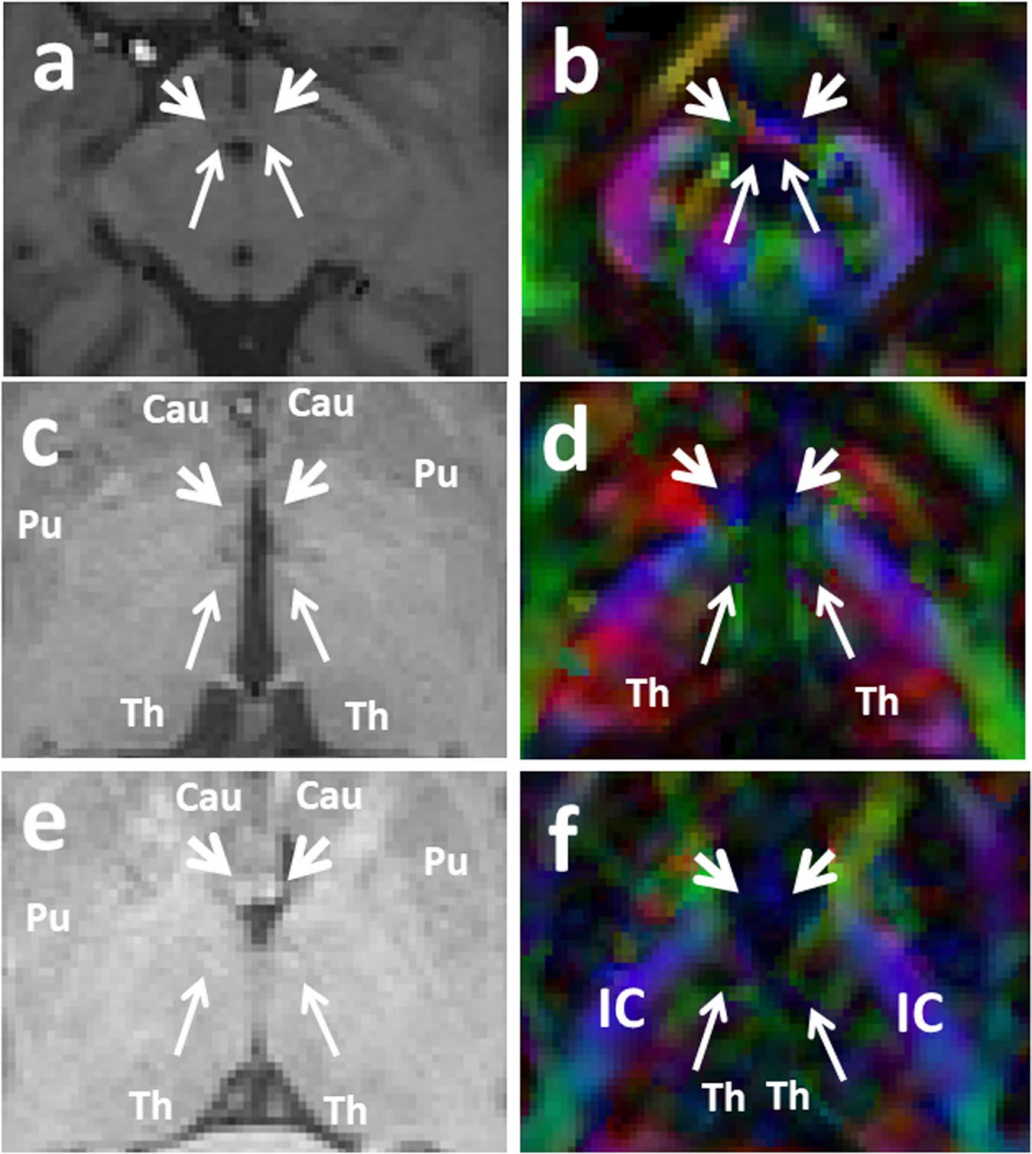
Three successive color-coded DTI map and corresponding T1 weighted MRI sequences in axial planes from caudal to cranial demonstrating the relationship of the postcommissural fibers of the fornix and stria terminalis (pointed by short arrows), with the MTT (pointed by long arrows) as they course parallel to each other. Four bright spots are visible on the T1 weighted images at the level of mammillary bodies and higher levels (**a**,**c**,**e**). The two frontal bright spots represent the forniceal columns. The two dorsal bright spots represent the MTT on both sides (Fig. **a**,**c**,**e**). As it’s visible on Fig. 3a,b, the ascending fibers of the MTT arise from the mammillary bodies (MB), course posteriorly and then cranially behind the poscommissural fibers of the fornix and stria terminalis (pointed by short arrows). The MTT then rises within the anterior thalami (Th) projecting more laterally as its visible on the (**e** and **f**). The MTT fibers then terminate in the anterior and medial thalamic nuclei in each side. Cau = caudate head; IC = internal capsule; Pu = putamen; Th = thalamus.

**Figure 4:**
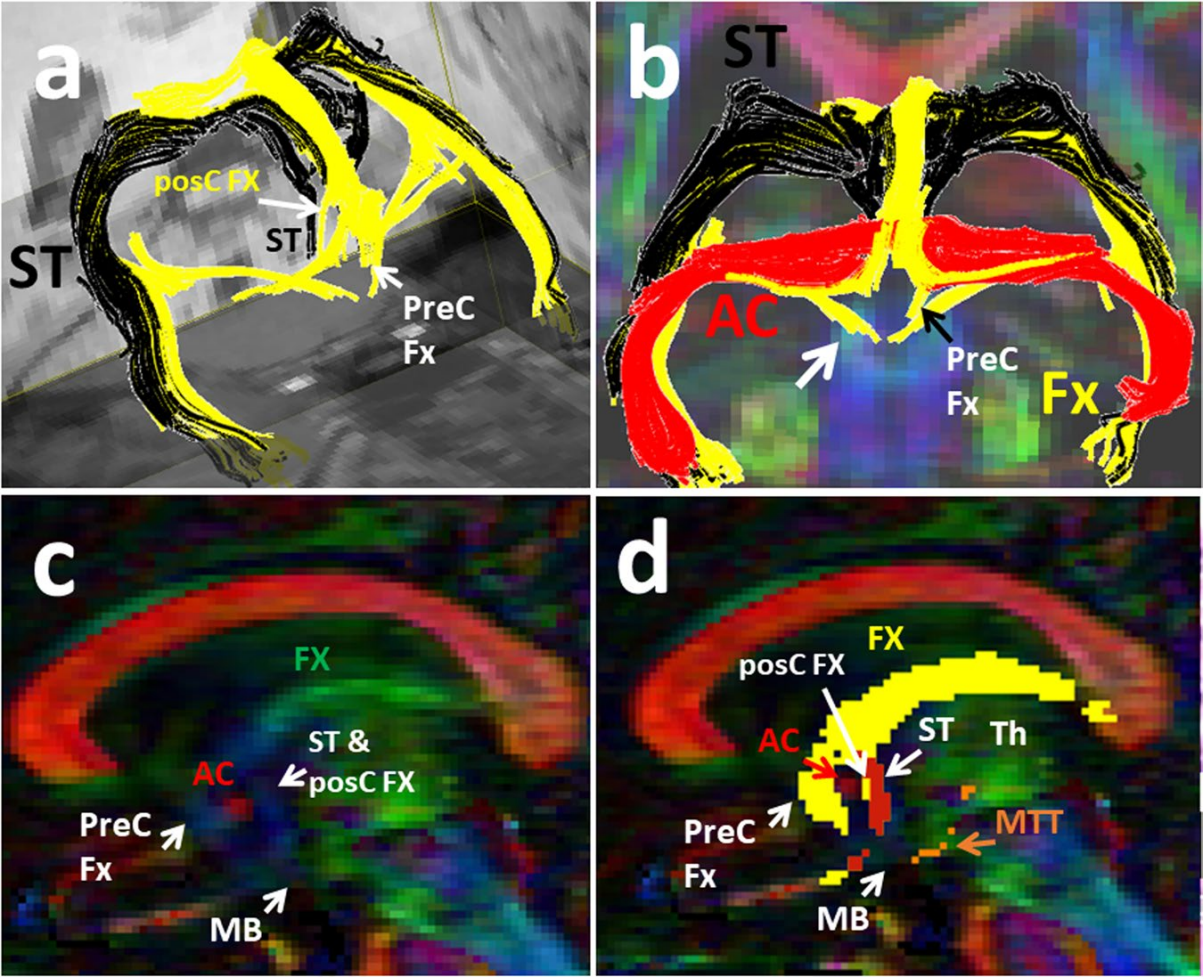
Three-dimensional reconstructions of the fornix (in yellow) and the stria terminalis (in black, **a** and **b**). The vertical blue fibers anterior to the anterior commissure (AC) shown in c, are the precommisural (PreC) fibers of the fornix (Fx). The vertical blue fibers posterior to the anterior commissure (AC) shown in c, are a combination of the postcommissural (PosC) fibers of the fornix and stria terminalis (ST). The corresponding trajectory of the fornix (yellow) and stria terminalis (shown in red) are shown in d. The precommissural (PreC) and postcommissural (PosC) fibers of the fornix are shown in yellow color anterior and posterior to the AC respectively. The trajectory of the MTT is shown (in orange in d) arising from the mammillary bodies (MB) and coursing posteriorly and then cranially toward the thalamus (Th).

**Figure 5:**
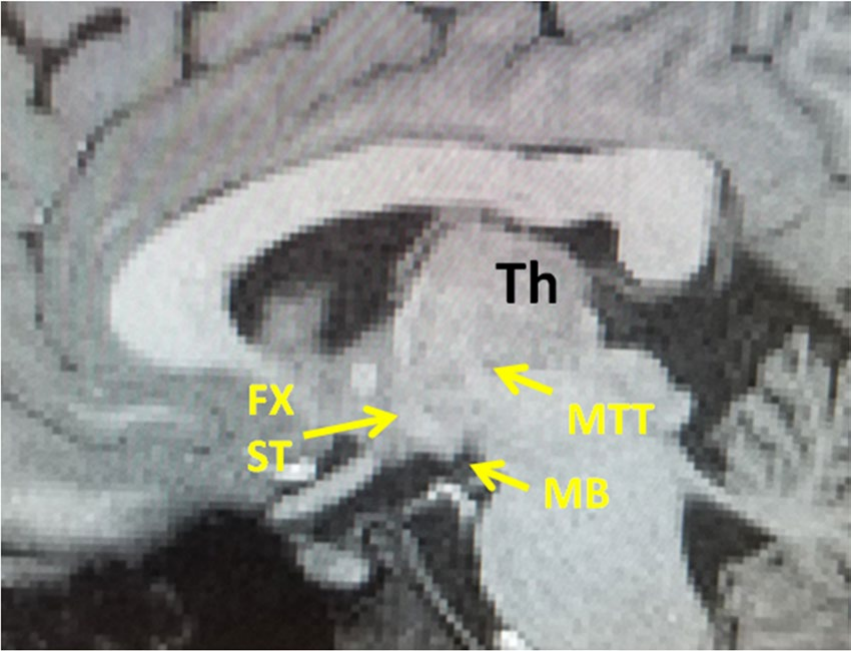
Sagittal T1 weighted MRI showing the trajectory of the MTT in relation with the precommissural fibers of the fornix and stria terminalis (Fx, ST). The MTT is visible by a T1 hypointense tract seen arising from the mammillary body (MB), projecting posteriorly and then cranially within the thalamus. The superior aspect of the MTT is not visible in this sagittal plane since the MTT projects more laterally as it ascends within the thalamus. The postcommissural fibers of the fornix and stria terminalis are visible by a T1 hypointense bundle vertically arising from the mammillary bodies.

**Figure 6:**
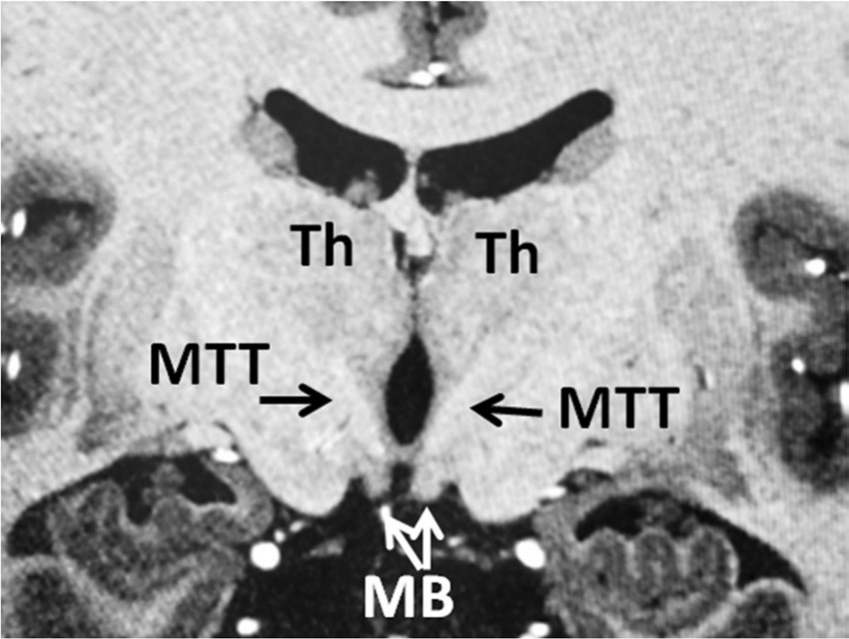
Coronal T1 weighted MRI showing the trajectory of bilateral MTTs on the coronal level just posterior to the mammillary bodies (MB). The bilateral MTTs are visible by T1 hypointense tracts coursing cranially toward the thalamus (Th) after exiting from the mammillary bodies. Please attention that the MTT already projected posteriorly before reaching this coronal level where it ascends into the thalamus. Lateral bendings of the MTTs are visible on this plane as they ascend within the thalami.

The trajectory of the mammillothalamic tract and the anatomical parcellations of the MTT in relation to the forniceal columns and stria terminalis are demonstrated in the present study (Figs 3–5). The MTTs are identified bilaterally in all subjects. A common pattern of this tract is observed bilaterally among the fifteen subjects. The high-resolution diffusion tensor tractography also revealed more detailed anatomical information regarding the mammillothalamic tract such as the lateral curvature and anatomical connections (Figs 2–5). Anatomical parcellations and illustrations of the mammillothalamic tract in relation to the fornix and stria terminalis are also demonstrated in the axial planes (Fig. 3). Four bright spots are visible on the T1 weighted images at the level of mammillary bodies and higher levels (Fig. 3a,c,e). The two frontal bright spots represent the forniceal columns. The two dorsal bright spots represent the MTT on both sides (Fig. 3a,c,e). We also demonstrate the tractography of the precommissural and postcommossural fibers of the forniceal columns along with stria terminalis, which course parallel to the MTT and connect to the region of mammillary bodies as well (Figs 2, 4d and 5). We further demonstrate the trajectories of the MTT, postcommissural fibers of the fornix and stria terminalis on the high spatial resolution T1 weighted anatomical MRI and their relationship with one another (Figs 5, 6).

### The mammillothalamic tract

The mammillothalamic is a projection bundle that arises from the mammillary bodies. Unlike the postcommissural fibers of the fornix and stria terminalis which rise cranially from the mammillary bodies (Figs 3, 4), the MTT projects dorsally first and then cranially toward the thalamus. In this course, the MTT rises just lateral to the third ventricle (Fig. 6) to reach the inferior aspects of the thalami (Figs 2–6). The mammillothalamic tract then projects laterally within the anterior thalamus demonstrating a lateral curvature within the thalamus (Figs 2, 3, 6). The fibers of the MTT then terminate in the anterior thalamic nuclei (Fig. 3e,f).

## Discussion

To the best of our knowledge, the current study is the first to thoroughly depict the trajectory of the mammillothalamic tract and its relationship with adjacent forniceal columns and stria terminalis using high spatial resolution DTI data. The mammillothalamic tract arises from cells in both the medial and lateral nuclei of the mammillary bodies and by fibers that are directly continued from the fornix according to the atlas of anatomy^1^. These fibers connect the mammillary bodies to the anterior thalamic nuclei^1,25–31^. A recent tractography study by Kwon *et al*. attempted to trace the mammillothalamic tract^32^. However, the trajectory of the MTT illustrated by these authors appears to disagree with our result and known detailed anatomy of the MTT. In the study by Kwon *et al*., the MTT showed an anterior reflection and convexity of the fiber tract. Our results showed a different pattern of the tract, with a lateral reflection and convexity of the MTT fibers, which coincide with the known connections of these tracts from the atlas of anatomy^1^ and prior animal studies^31,33,34^. The MTT arises from the mammillary bodies, which are anatomically located inferiorly and ventrally in relation to the thalamus. The MTT fibers project posteriorly and cranially just lateral to the third ventricles to the level of the thalami and then bend laterally within the anterior and medial aspects of the thalami which explain the lateral convexity of this tract (Figs 2a,b, 3c–f, 6). Our tractography result is in line with the result of the 3D reconstruction of the MTT based on the raw anatomical MRI data by Mori and Aggarwal, 2014^23^. Studies on the development of the mammillothalamic tract in rats also showed that the MTT grows in a rostrodorsal direction until it reaches the anterior thalamic nuclear group^31,34^.

The hypothalamus receives numerous sensory inputs, controls the autonomic nervous system and pituitary function and communicates with other parts of the limbic system, which is tightly connected to the prefrontal cortex. The human hypothalamus consists of a group of small nuclei located inferior and ventral to the thalamus and is subdivided into three regions: the supraoptic region, tuber cinereum, and the mammillary bodies. Projections to areas rostral to the hypothalamus including the septal nuclei are carried by the fornix, stria terminalis and amygdalofugal tract which have been traced in our recent DTI tractography studies^17,18^. The anterior thalamic nuclei receive projections from the fornix and mammillothalamic tract and connect to the orbitofrontal and anterior cingulate cortices via the anterior thalamic radiations. The more medially oriented fibers of the anterior thalamic radiations projecting to the prefrontal cortex were highlighted in our prior study as the prefronto-caudo-thalamic tract or inferior thalamic peduncle^35^.

As early as the 1900s, researchers conducted animal studies to map out the pathway of the MTT. Studies on rats, rabbits, and guinea pigs showed that the MTT originates in the lateral and medial mammillary nuclei, projects anterodorsally, and terminates in each of the three anterior thalamic nuclei: anteromedial, anteroventral, and anterodorsal^25–28,36^. Later, autoradiographic studies on rats and guinea pigs confirmed this pathway^29,36^. However, unlike the early studies claiming the medial mammillary nucleus projects into all three anterior thalamic nuclei, the autoradiographic studies indicated that only the anteromedial and anteroventral thalamic nuclei received projection from the medial mammillary nucleus. On the other hand, the lateral mammillary nucleus projects into the anterodorsal thalamic nucleus^29,36^. Further mapping of the MTT on rats and monkeys supported these findings^30,31,37^.

Researchers performed numerous studies to assess how surgically induced lesions on the MTT impacted memory and behavior in animals. Investigators have conducted experiments on the acquisition of cognitive tasks in rats undergoing MTT tractotomy. The results suggested that rats with MTT deficiencies have difficulty in acquiring new skills^4,5^. Researchers hypothesized that lesions of the MTT impair the acquisition of conditional discrimination involving spatial and visual contexts^6–8^. Furthermore, Thomas and Gash showed that complete transection of the mammillothalamic tract in rats resulted in substantial impairment in the ability to discriminate objects based on representational memory^38^. Besides its role in memory and behavior, MTT lesions in guinea pigs protected them from the convulsant and lethal actions of drug pentylenetetrazol thereby suggesting that the mammillothalamic tract may also be involved in the propagation and initiation of generalized seizures^16^. In addition to animal studies, human studies have shown that pathological changes to the MTT in humans result in memory impairment and amnesia^2,39^. While the memory impairment in these case reports could also be attributed to the extension of damage to the anterior thalamus, new case reports have since shown amnesia in patients despite no such damage to the anterior thalamus^40,41^. Studies indicate that damage to the MTT due to anterior-medial thalamic stroke causes severe memory impairment resulting in thalamic amnesia^42^. In addition, damage to the MTT after a thalamic infarct has been shown to be the only indicator of whether amnesia will develop in these patients^2,3,15^. A most recent study introduced the MTT as a new target for treatment of dementia and epilepsy^43^.

Our tractography results confirmed that the MTT is a solid white matter structure in the brain consisting of a band of fibers running along and parallel to the forniceal columns and anterior bodies serving as a major direct communication pathway of the thalamus with the mammillary bodies of the hypothalamus. We also demonstrated for the first time the close proximity of the MTT with the forniceal columns and stria terminalis (Figs 2–5). Furthermore, we presented, for the first time, the anterior arms of the fornix which serve as a direct ventral projection from the fimbria of the fornix and hippocampus toward the hypothalamus (white arrow in Fig. 4b). These findings add to our knowledge of the extensive and complex connectivity of the mammillary region of the hypothalamus. In particular, the local direct connections we have demonstrated of the hippocampus and fornix with the mammillary body nuclei indicate the existence of significant local circuits as well as more distant circuits of the hypothalamic nuclei such as with the amygdala via the fornix, stria terminalis, and the amygdalofugal tracts^17,18^. Our recent results regarding the tractography findings of the stria terminalis and amygdalofugal tract^17,18^ were validated by a most recent ultra-high spatial resolution (isotropic 0.25 mm resolution) postmortem human study^44^. The most recent ultra-high spatial resolution postmortem study by Mori *et al*. did not address the tractography of the MTT in the human brain. However, another high spatial resolution study by Mori and Aggarwal, 2014, demonstrated the 3D reconstruction of the MTT based on the row anatomical data which corroborates with and validates the current results of the MTT tractography^23^. Our results may be further validated by future postmortem studies with focusing on the delicate thalamo-limbic connections.

A given DTI-based trajectory may not correspond exactly to the fine distribution of actual axonal bundles due to crossing and kissing fibers that may cause false positive or false negative results^45,46^. However, our observations were consistent in all subjects which renders false positive results very unlikely. In our experience, the main challenge on the way of tracking the mammillothalamic tract was marked crossing with the major anterior-posteriorly oriented fibers such as the anterior thalamic radiations and the prefronto-caudo-thalamic pathways which were traced in our past DTI tractography study^35^. Since the MTT courses side by side with the stria terminalis and fornix, kissing fiber phenomenon may also result in fiber switch (false positive result) or abrupt termination of the tracking algorithm (false negative result)^45^. This confusion may be the source of some false positive results in studies that used a lower spatial resolution^32^ due to partial volume averaging and heavy crossing fibers in the region of the MTT. This further emphasizes that tractography results should be carefully interpreted in the context of well-established anatomical data known by the atlas of anatomy and prior dissection studies^43–45^. To avoid this confusion we also traced the fornix and stria terminalis on each side to better orient the readers to the close proximity of these white matter bundles (Fig. 4). In our experience, the ability to trace the major adjacent fine fiber tracts such as the fornix and stria terminalis^18^ was a valuable factor in our success to distinguish these convoluted bundles from one another. Using high spatial resolution DTI data increased the detectable anisotropy within the gray matter structures and helped to trace the thin white matter fibers passing through the hypothalamic and thalamic nuclei^35^. The complexity of the fibers within a voxel remains to be a limitation of the diffusion tensor tractography model. Inability to distinguish between the efferent and afferent fiber tracts is also another limitation of DTT technique. The tractography of the MTT may not be feasible in brain pathology such as high grade thalamic glioma with significant adjacent edema due to drop of anisotropy which is another limitation of diffusion tensor model. Future studies using high magnetic field MRI, higher angular diffusion technique or postmortem exams with focusing on the MTT may further validate our results^23,43,47^.

A precise understanding of the limbic white matter anatomy is essential for unraveling the complex functional network of the limbic system. This study may be one step forward toward unravelling the complex neuronal network of the limbic circuitry and to advance our understanding of limbic function and numerous limbic related disorders such as schizophrenia, depression, OCD, and anxiety disorder among other pathologies.

## Conclusions

In this report, we demonstrated the detailed trajectory of the mammillothalamic tract using a high spatial resolution diffusion tensor deterministic tractography technique at 3 T. We also depicted the relationship of the MTT with adjacent forniceal columns. Our tractography results are supported by the atlas of anatomy^1^ as well as prior animal and human studies^23,25–28,36^.
